# Supplementary material for: Cross-National variations in mental health: A cross-sectional study on depression, anxiety, and stress among university staff and students in Sub-Saharan African
Source: PLoS One. 2025 Jun 3;20(6):e0322163. doi: 10.1371/journal.pone.0322163 (PMC12133007; doi:10.1371/journal.pone.0322163)
Supplement: Attachment — Submitted filename: PONE-D-24-56128_reviewer.pdf [file pone.0322163.s001.pdf]

PLOS ONE

Cross-National Variations in Mental Health: A Cross-sectional Study on Depression, Anxiety, and Stress in sub-Saharan Africans

--Manuscript Draft--

**Manuscript Number:**	PONE-D-24-56128
**Article Type:**	Research Article
**Full Title:**	Cross-National Variations in Mental Health: A Cross-sectional Study on Depression, Anxiety, and Stress in sub-Saharan Africans
**Short Title:**	Variations in Mental health conditions by SSA countries
**Corresponding Author:**	Uchechukwu L. Osuagwu, Ph.D; MSc, OD<br>Western Sydney University - Campbelltown Campus<br>Campbelltown, New South Wales AUSTRALIA
**Keywords:**	Mental health; Depression; Anxiety; Stress; sub-Saharan Africa; Education; Malawi; Nigeria; Ghana; Mozambique
**Abstract:**	Purpose<br><br>Mental health disorders are global concerns, but their impact varies across regions. In sub-Saharan Africa (SSA), the influence of country-specific factors on mental health is under-researched. This study investigates the influence of country of origin on the prevalence and severity of mental health conditions among university students and staff across select SSA countries.<br><br>Method<br><br>A cross-sectional, web-based survey using the Depression, Anxiety, and Stress Scale-21 (DASS-21) was conducted from 16 April to 18 November 2024. The survey was distributed online through multiple African social networks, reaching students and staff from different universities across Africa. Prevalence estimates for anxiety, depression, and stress were based on binomial distribution with Clopper-Pearson confidence intervals, while country-level differences were assessed using univariate odds ratios and multiple logistic regression.<br><br>Results<br><br>Of the 3227 participants, aged 25.3±8.6 (mean±SD), the majority (1850, 57.3%) were females. Findings revealed that Nigerians reported the highest prevalence of severe and extremely severe mental health conditions, while Ghanaians recorded the lowest levels across all mental health categories. Multivariable analysis revealed that, compared to Ghanaians, respondents from Malawi had the strongest odds for mental health challenges followed by Mozambique and Nigeria. The adjusted odds ratios (AORs) for Malawi were 4.39 (95% CI: 3.28 – 5.89), 3.86 (95% CI: 2.81 – 5.29) and 4.51 (95% CI: 3.33 – 6.11) for depression, anxiety, and stress, respectively.<br><br>Conclusions<br><br>This study found significant differences in mental health outcomes between participants from Ghana, Malawi, Mozambique, and Nigeria. Respondents from Malawi had the greatest risk for depression, anxiety, stress, and combinations of those conditions suggesting that socioeconomic factors, such as inadequate healthcare infrastructure, play a key role in the mental health of this population. In contrast, participants from Ghana reported the lowest rates of mental health issues across the board, potentially because that country has stronger social support systems and mental health policies. Many participants reported experiencing more than one mental health issue, indicating a need for better mental healthcare resources in SSA.
**Order of Authors:**	Oforbuike Onyebuchi Ike, OD
	Michael Agyemang Kwarteng, MPH. OD

Powered by Editorial Manager® and ProduXion Manager® from Aries Systems Corporation

	Grace Ogbonna, PhD, OD
	Isaura Brito dos Santos, BSc, MPH
	Osamudiamen McHillary Ogiemudia, OD
	Anayochukwu E Anyasodor, MPH. PHD
	Ellen Konadu Antwi-Adjei, OD
	Okechi Ulumma Amaechi, Professor
	Ejididke Gertrude Ebele, PHD. OD
	Ngozika Esther Ezinne, MPH. OD
	Khathutshelo Percy Mashige, Professor
	Antor Ndep, DrPH
	Edith Daniel-Nwosu, PhD, OD
	Bernadine Nsa Ekpenyong, PhD, MPH, OD
	Tshubelela Sello Simon Magakwe, Professor
	Kingsley E Agho, PhD, MPH
	Ayomikun Nifemi Dahunsi, MPH. OD
	Oyelola A Adegboye, PhD
	Kelechi C Ogbuehi, Professor
	Uchechukwu L. Osuagwu, Ph.D; MSc, OD

Additional Information:	
**Question**	**Response**
**Financial Disclosure**<br><br>Enter a financial disclosure statement that describes the sources of funding for the work included in this submission. Review the submission guidelines for detailed requirements. View published research articles from *PLOS ONE* for specific examples.<br><br>This statement is required for submission and **will appear in the published article** if the submission is accepted. Please make sure it is accurate.	The author(s) received no specific funding for this work.

**Funded studies**<br>Enter a statement with the following details:<br>• Initials of the authors who received each award<br>• Grant numbers awarded to each author<br>• The full name of each funder<br>• URL of each funder website<br>• Did the sponsors or funders play any role in the study design, data collection and analysis, decision to publish, or preparation of the manuscript?<br><br>Did you receive funding for this work?	
**Competing Interests**<br><br>Use the instructions below to enter a competing interest statement for this submission. On behalf of all authors, disclose any competing interests that could be perceived to bias this work—acknowledging all financial support and any other relevant financial or non-financial competing interests.<br><br>This statement is required for submission and **will appear in the published article** if the submission is accepted. Please make sure it is accurate and that any funding sources listed in your Funding Information later in the submission form are also declared in your Financial Disclosure statement.<br><br>View published research articles from *PLOS ONE* for specific examples.	The authors have declared that no competing interests exist.

Powered by Editorial Manager® and ProduXion Manager® from Aries Systems Corporation

**NO authors have competing interests**<br><br>Enter: *The authors have declared that no competing interests exist.*<br><br>**Authors with competing interests**<br><br>Enter competing interest details beginning with this statement:<br><br>*I have read the journal's policy and the authors of this manuscript have the following competing interests: [insert competing interests here]*<br><br>* typeset	
**Ethics Statement**<br><br>Enter an ethics statement for this submission. This statement is required if the study involved:<br><br>• Human participants<br>• Human specimens or tissue<br>• Vertebrate animals or cephalopods<br>• Vertebrate embryos or tissues<br>• Field research<br><br>Write "N/A" if the submission does not require an ethics statement.<br><br>General guidance is provided below. Consult the submission guidelines for detailed instructions. **Make sure that all information entered here is included in the Methods section of the manuscript.**	The study was evaluated as posing minimal to no risk to participants and was conducted in full adherence to the ethical principles outlined in the 1967 Declaration of Helsinki. Ethical approval was obtained from several institutions, including the Research Ethics Committee of the Federal University of Technology Owerri, Nigeria (FUT/SOHT/REC/vol. 4/2), the Research Ethics Committee of Abia State University Uturu, Nigeria (ABSU/REC/OPT/002/2024), the Health Research Ethics Committee of Bayero University Kano, Nigeria (NHREC/BUK-HREC/476/10/2311) and the Committee on Human Research, Publication and Ethics, Kwame Nkrumah University of Science and Technology, Ghana (CHRPE/AP/374/24). Verbal informed consent was obtained electronically from all participants by asking them to respond with a "Yes" or "No" to the question, "Do you consent to voluntarily participate in this survey?" Consent was implied when participants selected the "Yes" option. To ensure this, the consent question was made mandatory, preventing participants from advancing in the questionnaire without responding. In addition, the university granted a waiver for obtaining assent due to the minimal risk associated with the study design.

**Format for specific study types**<br><br>**Human Subject Research (involving human participants and/or tissue)**<br>• Give the name of the institutional review board or ethics committee that approved the study<br>• Include the approval number and/or a statement indicating approval of this research<br>• Indicate the form of consent obtained (written/oral) or the reason that consent was not obtained (e.g. the data were analyzed anonymously)<br><br>**Animal Research (involving vertebrate animals, embryos or tissues)**<br>• Provide the name of the Institutional Animal Care and Use Committee (IACUC) or other relevant ethics board that reviewed the study protocol, and indicate whether they approved this research or granted a formal waiver of ethical approval<br>• Include an approval number if one was obtained<br>• If the study involved *non-human primates*, add *additional details* about animal welfare and steps taken to ameliorate suffering<br>• If anesthesia, euthanasia, or any kind of animal sacrifice is part of the study, include briefly which substances and/or methods were applied<br><br>**Field Research**<br><br>Include the following details if this study involves the collection of plant, animal, or other materials from a natural setting:<br>• Field permit number<br>• Name of the institution or relevant body that granted permission	
**Data Availability**<br><br>Authors are required to make all data underlying the findings described fully available, without restriction, and from the time of publication. PLOS allows rare exceptions to address legal and ethical concerns. See the PLOS Data Policy and FAQ for detailed information.	Yes - all data are fully available without restriction

A Data Availability Statement describing where the data can be found is required at submission. Your answers to this question constitute the Data Availability Statement and **will be published in the article**, if accepted.<br><br>**Important:** Stating 'data available on request from the author' is not sufficient. If your data are only available upon request, select 'No' for the first question and explain your exceptional situation in the text box.<br><br>Do the authors confirm that all data underlying the findings described in their manuscript are fully available without restriction?	
**Describe where the data may be found in full sentences. If you are copying our sample text, replace any instances of XXX with the appropriate details.**<br><br>• If the data are **held or will be held in a public repository**, include URLs, accession numbers or DOIs. If this information will only be available after acceptance, indicate this by ticking the box below. For example: *All XXX files are available from the XXX database (accession number(s) XXX, XXX.)*.<br>• If the data are all contained **within the manuscript and/or Supporting Information files**, enter the following: *All relevant data are within the manuscript and its Supporting Information files.*<br>• If neither of these applies but you are able to provide **details of access elsewhere**, with or without limitations, please do so. For example:<br><br>*Data cannot be shared publicly because of [XXX]. Data are available from the XXX Institutional Data Access / Ethics Committee (contact via XXX) for researchers who meet the criteria for access to confidential data.*<br><br>*The data underlying the results presented in the study are available from (include the name of the third party*	The data that support the findings of this study are available on request from the corresponding author, ULO.

and contact information or URL).<br>• This text is appropriate if the data are owned by a third party and authors do not have permission to share the data.<br><br>* typeset	
Additional data availability information:	Tick here if the URLs/accession numbers/DOIs will be available only after acceptance of the manuscript for publication so that we can ensure their inclusion before publication.

Powered by Editorial Manager® and ProduXion Manager® from Aries Systems Corporation

Manuscript

Click here to access/download;Manuscript;Main manuscript_Cross-National Differences in MH.docx

Original Article

Cross-National Variations in Mental Health: A Cross-sectional Study on Depression, Anxiety, and Stress in sub-Saharan Africans

[1]Oforbuike Onyebuchi Ike, [2]Michael Agyemang Kwarteng, [3]Grace Ogbonna, [4]Isaura Brito dos Santos, [5]Osamudiamen McHillary Ogiemudia, [6]Anayochukwu E. Anyasodor, [7]Ellen Konadu Antwi-Adjei, [8]Okechi Ulumma Amaechi, [9]Ejididke Gertrude Ebele, [10]Ngozika Esther Ezinne, [11]Khathutshelo Percy Mashige, [12]Antor Ndep, [13]Edith Daniel-Nwosu, [14]Bernadine Nsa Ekpenyong, [15]Tshubelela Sello Simon Magakwe, [16]Kingsley E. Agho, [17]Ayomikun Nifemi Dahunsi, [18]Oyelola A. Adegboye, [19]Kelechi C Ogbuehi, [20]*Uchechukwu Levi Osuagwu, [11]Center for eyecare and Public Health Intervention Initiative (CEPHII).

Authors Affiliations

[1]Department of Optometry, Bayero University Kano, PMB 3011, Gwarzo Road Kano, Nigeria. ioonyebuchi.opt@buk.edu.ng

[2]Optometry Unit, Department of Clinical Surgical Sciences, Faculty of Medical Sciences, The University of the West Indies, St Augustine Campus, Trinidad and Tobago. Michael.Kwarteng@uwi.edu

[3]Department of Optometry, Mzuzu University Malawi, Luwinga, P/Bag 201, Mzuzu. ogbonnagrace19@gmail.com

[4]Department of Community Health, Faculty of Medicine, Eduardo Mondlane University, Salvador Allende Ave, #702 Maputo, Mozambique. Isabrito462@gmail.com

[5]Department of Optometry, Faculty of Life Sciences, University of Benin, P.M.B 1154, Benin City, Nigeria; and Department of Optometry, College of Medical and Health Sciences, Novena University, Ogume, P.M.B. 2, Kwale, Delta State, Nigeria. mc_hillary@hotmail.com

[6]Rural Health Research Institute, Charles Sturt University, Orange, NSW 2800, Australia.aanyasodor@csu.edu.au

[7]Department of Optometry and Visual Science, College of Science, Kwame Nkrumah University of Science and Technology, P. V. Obeng Ave, P.O. Box 80404 Kumasi, Ghana. ekantwi-adjei@knust.edu.gh

[8]Department of Optometry, Faculty of Health Sciences, Abia State University, P.M.B 2000, Uturu, Abia State, Nigeria. okechi.amaechi@abiastateuniversity.edu.ng

[9]Department of Optometry, Madonna University, Nigeria, 510242, Elele, Rivers State. ejidikeebele@madonnauniversity.edu.ng

[10]Bathurst Rural Clinical School, Western Sydney University, Australia, Optometry Unit, Department of Clinical Surgical Sciences, Faculty of Medical Sciences, The University of the West Indies, St Augustine Campus, Trinidad and Tobago. ezinne.ngozi@gmail.com

[11]Department of Optometry, African Vision Research Institute, Private Bag X54001, Durban, 4000. mashigek@ukzn.ac.za

[12]Health Education & Health Promotion Unit, Department of Public Health, Faculty of Allied Medical Sciences, College of Medical Sciences, University of Calabar, Cross River State, Calabar 540271, Cross River State, Nigeria. drndep@gmail.com

[13]Department of Optometry, Federal University of Technology Owerri, PMB 1526 Owerri, Imo State, Nigeria. edithedfuto@gmail.com

[14]Department of Public Health, Faculty of Allied Medical Sciences, College of Medical Sciences, University of Calabar, Calabar 540271, Cross River State, Nigeria. bekpenyong@unical.edu.ng

[15]Disciple of Optometry, University of KwaZulu Natal, Durban, 4000, South Africa. sellomagakwe80@gmail.com

[16]School of Health Science, Western Sydney University, Campbelltown, NSW 2560, Australia. AND African Vision Research Institute, Discipline of Optometry, University of KwaZulu-Natal, Westville Campus, Durban 3629, South Africa. k.agho@westernsydney.edu.au

[17]Millenium Eye Center, University of Medical Sciences Teaching Hospital, Akure 340283, Ondo State. nihfeymih@gmail.com

[18]Menzies School of Health Research, Charles Darwin University, NT 0811, Australia. oyelola.adegboye@menzies.edu.au

[19]Department of Medicine, Dunedin School of Medicine, University of Otago, Dunedin 9054, New Zealand. kelechi.ogbuehi@otago.ac.nz

*[20]Bathurst Rural Clinical School (BRCS), School of Medicine, Western Sydney University, Bathurst, NSW 2795, Australia

*Corresponding author: Uchechukwu. L. Osuagwu; email: l.osuagwu@westernsydney.edu.au; Tel.: +61-4011-93-234,

https://orcid.org/0000-0002-1727-6914

[P] These authors contributed equally to this work

Acknowledgments: The authors acknowledge the support of the centre for Eye Care and Public Health Intervention Initiative (CEPHII) during data collection and thank the following individuals for their contribution during review: Aiyshetu Shuaibu, Godwin O Ovenseri-Ogbomo, Isaac Koomson, and Haile Woretaw.

Keywords: Mental health, depression, anxiety, stress, sub-Saharan Africa, Education, Malawi; Nigeria, Ghana, Mozambique.

Abstract

Purpose: Mental health disorders are global concerns, but their impact varies across regions. In sub-Saharan Africa (SSA), the influence of country-specific factors on mental health is under-researched. This study investigates the influence of country of origin on the prevalence and severity of mental health conditions among university students and staff across select SSA countries.

Method: A cross-sectional, web-based survey using the Depression, Anxiety, and Stress Scale-21 (DASS-21) was conducted from 16 April to 18 November 2024. The survey was distributed online through multiple African social networks, reaching students and staff from different universities across Africa. Prevalence estimates for anxiety, depression, and stress were based on binomial distribution with Clopper-Pearson confidence intervals, while country-level differences were assessed using univariate odds ratios and multiple logistic regression.

Results: Of the 3227 participants, aged 25.3±8.6 (mean±SD), the majority (1850, 57.3%) were females. Findings revealed that Nigerians reported the highest prevalence of severe and extremely severe mental health conditions, while Ghanaians recorded the lowest levels across all mental health categories. Multivariable analysis revealed that, compared to Ghanaians, respondents from Malawi had the strongest odds for mental health challenges followed by Mozambique and Nigeria. The adjusted odds ratios (AORs) for Malawi were 4.39 (95% CI: 3.28 – 5.89), 3.86 (95% CI: 2.81 – 5.29) and 4.51 (95% CI: 3.33 – 6.11) for depression, anxiety, and stress, respectively.

Conclusions: This study found significant differences in mental health outcomes between participants from Ghana, Malawi, Mozambique, and Nigeria. Respondents from Malawi had the greatest risk for depression, anxiety, stress, and combinations of those conditions suggesting that socioeconomic factors, such as inadequate healthcare infrastructure, play a key role in the mental health of this population. In contrast, participants from Ghana reported the lowest rates of mental health issues across the board, potentially because that country has stronger social support systems and mental health policies. Many participants reported experiencing more than one mental health issue, indicating a need for better mental healthcare resources in SSA.

Introduction

Mental health disorders, including depression, anxiety and stress, are a growing public health concern, with significant global prevalence [1, 2] which varies across populations[3, 4]. For example, the global incidence of depression increased from 172 million cases in 1990 to 258 million in 2017, marking a 49.86% rise [5, 6]. According to the World Health Organization[7], approximately 280 million people worldwide suffer from depression, a condition that disproportionately affects women and is often linked to economic burdens, stigma, discrimination, and social exclusion[8, 9]. Mental health disorders affect individuals across all ages, genders, and socioeconomic backgrounds, exerting a widespread impact across diverse geographic and demographic groups[10].

Furthermore, the population differences in mental health outcomes highlight the country-specific variations that can exacerbate the burden of mental health disorders. For instance, in low- and middle-income countries, common barriers to improved mental healthcare include the low prioritisation of mental healthcare, fragmented services, a shortage of mental health professionals, and inadequate leadership within public health systems[11, 12]. Moreover, students and staff in universities within under-resourced educational systems in low- and middle-income countries are especially susceptible to mental health challenges, as they contend with distinctive stressors such as heavy academic workloads and intense pressure to perform[13-15]. Researching these groups offers valuable insights into their mental health challenges, enabling targeted interventions to improve well-being, academic performance, and workplace productivity, with benefits extending to society at large. Understanding these variations facilitates the development of targeted strategies that address the unique needs and challenges faced by different populations.

Previous research has predominantly focused on mental health disorders within individual countries; however, there remains a lack of studies examining the influence of the country of origin on mental health outcomes, particularly regarding depression, anxiety, and stress. The current body of

literature frequently neglects the role of cross-cultural differences among countries, leading to a significant gap in understanding. This study, therefore, aimed to conduct a cross-national comparison in SSA to assess the influence of country of origin on the prevalence and severity of mental health conditions, specifically depression, anxiety, and stress. The objectives of this study include determining the prevalence of depression, anxiety, and stress across different countries, identifying factors contributing to variations in mental health outcomes, and assessing the relationship between these factors, country of origin, and the prevalence and severity of mental health conditions, particularly in relation to depression, anxiety, and stress.

Materials and Methods

Participants

The participants in this study included students and staff affiliated with various universities and colleges across several sub-Saharan African (SSA) countries, predominantly from Nigeria, Ghana, Malawi, and Mozambique. Individuals associated with African institutions were targeted for recruitment. Before participation, all potential participants were provided with detailed information about the study, including its nature and purpose, through an online preamble.

Study design

This study employed a web-based, cross-sectional survey [Supplementary File 1], utilizing a convenience sampling method. The data collection took place from Tuesday, April 16th to Monday, November 18, 2024, using a validated questionnaire. The questionnaire was translated and administered in Portuguese and English languages via Google Forms, across SSA countries. The inclusion of a Portuguese version was intended to facilitate participation from non-English speaking individuals in Lusophone countries, addressing limitations noted in previous surveys [16, 17].

To ensure efficient distribution and accessibility, an electronic link to the survey was shared via social media platforms, including WhatsApp and Facebook, as well as through the authors' email contacts. The survey distribution primarily utilized a snowballing approach, leveraging virtual networks to reach individuals who engaged with social media and other online platforms. This method was both time- and cost-effective for data collection [18-20]. The use of an online survey facilitated a broad outreach to a large cohort across SSA, enabling timely data collection with limited resources. Moreover, the use of social media platforms significantly allowed for the rapid dissemination of survey information [21-23].

Questionnaires

Two validated, self-administered surveys were adapted, with minor modifications, to align with the objectives of this study. The first section of the survey collected data on the participants' sociodemographic characteristics, including age, gender, country of origin, institutional affiliation, location, year of study, department, and name of school. Students were asked to indicate their course of study, while staff reported the school in which they were employed. Additional variables included the highest level of educational qualification, marital status, and faculty designation (i.e., student, academic, or non-academic staff). The reporting of this study strictly adhered to the STrengthening the Reporting of OBservational studies in Epidemiology (STROBE) guidelines for cross-sectional studies (Supplementary File 2)

DASS-21 Survey scale

This is a short version of the Depression, Anxiety, and Stress Scale-21 (DASS-21), utilized for psychological screening purposes [24]. The instrument, which has been previously validated [25-27] and pretested in this study, is specifically designed to distinguish between symptoms of depression, anxiety, and stress. Each of the three DASS-21 scales consists of 7 items, grouped into subscales with similar content. Responses were scored using a 4-point Likert scale ranging from 0 to 3, where 0 indicates "did not apply to me at all", 1 indicates "applied to me to some degree", 2 indicates "applied to me to a considerable degree", and 3 indicates "applied to me very much". In the assessment of

depression, a score of 0–9 on the depression subscale was classified as normal, 10–13 as mild, 14–20 as moderate, 21–27 as severe, and scores of 28 or higher as extremely severe. For anxiety, a score of 0–7 was considered normal, 8–9 as mild, 10–14 as moderate, 15–19 as severe, and 20 or above as extremely severe. Regarding stress, scores of 0–14 were classified as normal, 15–18 as mild, 19–25 as moderate, 26–33 as severe, and scores 34 and above as extremely severe.

The questionnaires were pre-tested one week before the main data collection on a sample of 30 participants, who were not included in the final survey. Following the pre-test, the questionnaire was revised based on the feedback received. The Cronbach's alpha (α) coefficient for the DASS-21 scale was determined to be 0.82, indicating good internal consistency across all items.

Inclusion and exclusion criteria

Survey responses from individuals who were not originally from SSA but were currently studying or working in the region were excluded from the analysis. Additionally, responses originating from identical Internet Protocol addresses and exhibiting similar demographic characteristics, such as age and gender, were considered duplicate entries. In such cases, only the most complete entry was retained for analysis. Participants included in the descriptive statistics were those who were studying or employed at tertiary institutions in SSA, had provided informed consent, and were currently residing in SSA. However, for the final analysis assessing factor significance, only data from participants who completed the entire survey including all items on the DASS-21 were included.

Sample size determination

The desired sample size was calculated [28] considering a precision of level of 3.5%, non-response rate of 10%, 95% confidence level, and proportion of adults with mental health as 50% since a similar cross-national study has not been conducted.

$$n = \frac{Z^2(1-P)}{\varepsilon^2 P}$$

This yielded a sample size of 3484 participants. This sample size was considered sufficient to detect statistically significant differences in the analysis of an online cross-sectional study investigating mental

health symptoms among university staff and students in SSA. However, a total of 3227 (92.6%) responses were included in the analysis.

Outcome variables

The study employed the DASS-21 survey instrument to measure three outcome variables: depression, anxiety, and stress, which were treated as categorical variables. These categorical variables were analyzed according to their respective classification thresholds. Additionally, mental health conditions were categorized based on their severity into dichotomized binary categories of 'Yes' or 'No'. For this, a depression score of 0-9 was classified as "No depression" and a score of 10+ as "Major depression". For anxiety, scores of 0-7 indicated "No anxiety" and 8+ indicated "Anxiety". For stress, scores of 0-14 were considered "No stress," while 15+ indicated "Stress". (Yes).

Predictor Variables

The following countries of origin in SSA were considered predictor variables due to having a big enough sample size: Ghana, Nigeria, Malawi, and Mozambique.

Covariates

The sociodemographic variables considered in the analysis included age, gender, marital status, educational attainment, faculty category (student, academic staff, and non-academic staff), religious affiliation, level of study, rurality, and country of residence.

Data analysis

The sample characteristics were presented using frequencies and percentages. Data from other participating countries were excluded from the analysis due to the limited number of respondents. Only countries with a minimum of 300 respondents were included in the analysis. The prevalence of anxiety, depression, and stress were estimated using the binomial distribution with Clopper-Pearson confidence intervals. To assess country-level differences in risk of anxiety, depression, stress, and their

combination, odds ratios (ORs) were calculated using univariate analysis to examine associations with the country of residence. Multivariable logistic regression was subsequently performed to investigate these associations further. The multivariable analysis was adjusted for the following variables: age group, gender, marital status, educational level, ethnicity, religion, location of workplace, and having children. This approach accounted for the influence of these factors on the relationship between country of origin and mental health outcomes. Adjusted odds ratios (AORs) and their 95% confidence intervals (CIs) were derived from the adjusted multivariable logistic regression model to quantify the relationships. Interaction terms were not included in the model due to the complexity of the data. Statistical significance was assessed at a 5% significance level. All statistical analyses were performed using Statistical Package for the Social Sciences (IBM Corp, SPSS Statistics for Windows Version 21.0, Armonk, NY, USA) and R version 4.0.3.

Ethical Consideration

The study was evaluated as posing minimal to no risk to participants and was conducted in full adherence to the ethical principles outlined in the 1967 Declaration of Helsinki. Ethical approval was obtained from several institutions, including the Research Ethics Committee of the Federal University of Technology Owerri, Nigeria (FUT/SOHT/REC/vol. 4/2), the Research Ethics Committee of Abia State University Uturu, Nigeria (ABSU/REC/OPT/002/2024), the Health Research Ethics Committee of Bayero University Kano, Nigeria (NHREC/BUK-HREC/476/10/2311) and the Committee on Human Research, Publication and Ethics, Kwame Nkrumah University of Science and Technology, Ghana (CHRPE/AP/374/24). Verbal informed consent was obtained electronically from all participants by asking them to respond with a "Yes" or "No" to the question, "Do you consent to voluntarily participate in this survey?" Consent was implied when participants selected the "Yes" option. To ensure this, the consent question was made mandatory, preventing

participants from advancing in the questionnaire without responding. In addition, the university granted a waiver for obtaining assent due to the minimal risk associated with the study design.

Results

Characteristics of the study population

The flowchart in Figure 1 illustrates the data process steps used in this study. After removing duplicates, non-African respondents, and countries with insufficient and incomplete data, a final sample of 3,221 participants was obtained, with the majority from Nigeria (1832, 56.9%) and Ghana (596, 18.5%). The mean±SD age of the participants was 25.3 ± 8.6 years (range, 15 to 72 years) (Table 1). The majority of respondents were students (n=2807, 87.0%) and females (1845, 57.3%). Most respondents identified as Christian (2691, 83.4%) and not married (2741, 85.1%), with nearly all respondents (96.4%) reporting African descent. Regarding educational attainment, 63.9% (n=2059) held Diploma and lower qualifications, while 36.1% (n=1162) had obtained a bachelor's degree and above. Additionally, 83.7% (n=2639) of respondents indicated that they did not have children. In terms of work location, 38.3% (n=1237) were employed in urban areas, although 36.8% of respondents had their residence unclassified.

Table 1: Distribution of socio-demographics by country of residence.

**Variables**	**Sub-group**	**Country of Residence, n (%)**				**N (%)**	**P-value**
		Ghana	Malawi	Mozambique	Nigeria		
Age group (years)	Younger age (<18yrs)	10 (0.3)	1 (0.0)	7 (0.2)	41 (1.3)	59 (1.8)	<0.001
	Youth (18-35yrs)	547 (17.0)	368 (11.4)	322 (10.0)	1552 (48.2)	2789 (86.6)	

	Adult and Elderly (≥36yrs)	39 (1.2)	28 (0.9)	67 (2.1)	239 (7.6)	373 (11.8)	
Gender, n=3197	Female	353 (11.0)	177 (5.5)	241 (7.5)	1,074 (33.6)	1,845 (57.7)	<0.001
	Male	236 (7.4)	218 (6.8)	151 (4.7)	747 (23.4)	1,352 (42.3)	
Ethnicity	African	583 (18.1)	395 (12.3)	347 (10.8)	1779 (55.2)	3104 (96.4)	<0.001
	Others	13 (0.4)	2 (0.1)	49 (1.5)	53 (1.6)	117 (3.6)	
Job description, n= 3192	Academic staff	40 (1.3)	16 (0.5)	49 (1.5)	198 (6.2)	303 (9.5)	<0.001
	Non-academic staff	23 (0.7)	7 (0.2)	11 (0.3)	47 (1.5)	88 (2.8)	
	Student	530 (16.6)	373 (11.7)	336 (10.5)	1568 (49.1)	2807 (87.9)	
Marital status	Married	35 (1.1)	51 (1.6)	75 (2.3)	319 (9.9)	480 (14.9)	<0.001
	Not married	561 (17.4)	346 (10.7)	321 (10.0)	1513 (47.0)	2741 (85.1)	
Religion	Christianity	526 (16.3)	380 (11.8)	324 (10.1)	1455 (45.2)	2691 (83.4)	<0.001
	Islamic	57 (1.8)	11 (0.3)	56 (1.7)	347 (10.8)	471 (14.6)	
	Others	13 (0.4)	6 (0.2)	16 (0.5)	30 (0.9)	65 (2.0)	
Highest level of education	Diploma and lower	430 (13.3)	337 (10.5)	244 7.6)	1048 (32.5)	2059 (63.9)	<0.001
	Bachelors and above	166 (5.2)	60 (1.9)	152 (4.7)	784 (24.3)	1162 (36.1)	
Do you have children, n=3152	Yes	51 (1.6)	54 (1.7)	108 (3.4)	300 (9.5)	513 (16.3)	<0.001
	No	535 (17.0)	330 (10.5)	292 (9.3)	1488 (47.2)	2639 (83.7)	

Work location	Urban	339 (10.5)	49 (1.5)	299 (9.3)	550 (17.0)	1237 (38.3)	<0.001
	Semi-urban	29 (0.9)	26 (0.8)	67 (2.1)	333 (10.3)	455 (14.1)	
	Rural	12 (0.4)	12 (0.4)	33 (1.0)	290 (9.0)	347 (10.8)	
	None of the above	216 (6.7)	310 (9.6)	3 (0.1)	659 (20.4)	1188 (36.8)	
Total		596 (18.5)	397 (12.3)	396 (12.3)	1832 (56.9)	3221 (100.0)	

Prevalence and distribution of severity of mental health conditions.

The prevalence of depression, anxiety, stress, and combinations of conditions—across four countries: Ghana, Malawi, Mozambique, and Nigeria varied significantly. Among the countries analyzed, participants residing in Malawi exhibited the highest prevalence of depression (72.3%, 95% CI: 67.6% – 76.6%) and anxiety (81.4%, 95% CI: 77.2% – 85.1%), respectively, whereas Ghana had the lowest at 55.2% (95% CI: 51.1% -59.2%). The overall prevalence of depression across all four countries was 53.3% (95% CI: 51.5% - 55.0%), while that of anxiety was 63.5% (95% CI: 61.8% - 65.2%). In terms of stress prevalence, participants living in Malawi also ranked the highest, with a rate of 50.6% (95% CI: 45.6% – 55.6%), and lowest in Ghana (20.6%, 95% CI: 17.5% - 24.1%), with an overall prevalence of 30.1% (95% CI: 28.6% -31.8%). For individuals experiencing at least two conditions, Malawi again showed the highest prevalence at 72.3% (95% CI: 67.6%-76.6%), and Ghana the lowest at 38.9% (95% CI: 35.0%-43.0%), with an overall prevalence of 51.2% (95% CI: 49.5%-52.9%). The prevalence of individuals experiencing all three mental health conditions was highest in Malawi at 45.3% (95% CI: 40.4%-50.4%) and lowest in Ghana at 17.3% (95% CI: 14.3-20.6), with an overall frequency of 27.3% (95% CI: 25.8-28.9). Mozambique and Nigeria exhibited elevated prevalence rates for co-occurring conditions and all three conditions, respectively, while Ghana consistently presented the lowest prevalence across most variables.

Figure 2 illustrates the distribution of respondents by their depression, anxiety, and stress levels, respectively. It shows that a significant proportion of respondents experienced moderate to severe levels of mental health disorders, particularly anxiety (Figure 2 top panel). Anxiety was predominantly categorized as 'extremely severe' in 27.6% of respondents (n = 893), with a lower percentage falling into the 'normal' anxiety category (n = 1175, 36.5%). For depression, about 23.0% had 'moderate' depression, while 15.5% reported 'mild' depression. In contrast, stress was largely classified as 'normal' (N = 2254, 69.9%), with relatively few individuals experiencing severe stress levels.

Figure 2 (bottom panel) shows the percentage of people experiencing mental health disorders by country of origin. Nigeria showed the highest percentage of participants experiencing severe and extremely severe mental health conditions across all categories. For depression and anxiety, a higher percentage of Nigerians reported severe (4.9% and 4.7%) and extremely severe (4.6% and 16.0%) depression, while Ghanaians reported notably lower proportions, particularly in the severe anxiety category (1.6%). For stress, Nigerian respondents made up the highest percentage of individuals experiencing severe (4.4%) and extremely severe stress (2.8%), while Ghanaians reported the lowest prevalence of severe (0.6%) and extremely severe stress (0.4%).

Univariate Analysis of factors associated with mental health condition by country of origin in SSA

Figure 3 indicates that, for depression, individuals in Malawi were 3.79 (95% CI: 2.89 to 5.00) times more likely to experience depression compared to those in Ghana. In comparison, those in Mozambique and Nigeria had odds ratios (OR) of 2.10 (95% CI: 1.62 to 2.72) and 1.57 (95% CI: 1.30 to 1.90), respectively. For anxiety, the odds were 3.54 (95% CI: 2.64 to 4.80) times higher in Malawi, 1.66 (95% CI: 1.28 to 2.17) times higher in Mozambique, and 1.30 (95% CI: 1.08 to 1.57) times higher in Nigeria. For stress, the odds in Malawi were 3.94 (95% CI: 2.99 to 5.23) times higher, in Mozambique 2.01 (95% CI: 1.51 to 2.68) times higher, and in Nigeria 1.49 (95% CI: 1.19 to 1.87) times higher. Regarding the likelihood of experiencing all three conditions simultaneously, Malawi

showed the highest odds at 3.97 (95% CI: 2.98 to 5.32), followed by Mozambique at 2.11 (95% CI: 1.56 to 2.85) and Nigeria at 1.68 (95% CI: 1.33 to 2.13). For experiencing any two conditions, the odds were 4.09 in Malawi (95% CI: 3.12 to 5.40), 2.04 in Mozambique (95% CI: 1.58 to 2.65), and 1.54 in Nigeria (95% CI: 1.27 to 1.85). Malawi consistently showed the highest odds of mental health conditions compared to Ghana, with ORs ranging from 3.54 to 4.09 for different mental health outcomes.

Multivariable Analysis of factors associated with mental health condition by country of origin in SSA

Table 2 presents the adjusted odds ratios (AORs) for mental health issues across the countries studied after adjusting for socio-demographic variables (See Table S1 for the full variable list). Malawi exhibited the highest risk, significantly surpassing the odds observed in Mozambique and Nigeria when compared to Ghana. Specifically, individuals in Malawi were 4.39 (95% CI: 3.28 – 5.89) times more likely to be depressed, 3.86 (95% CI: 2.81 – 5.29) more likely to experience anxiety, and 4.51 (95% CI: 3.33 – 6.11) times more likely to experience stress compared to respondents from Ghana. Additionally, individuals in Malawi were 4.67 (95% CI: 3.41 – 6.41) times more likely to experience all three conditions simultaneously and 4.64 (95% CI: 3.46 – 6.23) times more likely to face any two conditions. In Mozambique, the adjusted odds remained elevated (AOR=2.03, 95% CI: 1.55 – 2.67) for depression, 1.68 (95% CI: 1.27 – 2.22) for anxiety, and 1.98 (95% CI: 1.46 – 2.69) for stress. The likelihood of experiencing all three conditions was 2.08 (95% CI: 1.51 – 2.85) times higher, while the odds for any two conditions were 2.02 (95% CI: 1.54 – 2.66) in Malawi. Although the risk of mental health in Nigeria was lower than in Malawi and Mozambique, it still showed significant values, with AORs of 1.59 (95% CI: 1.30 – 1.95) for depression, 1.30 (95% CI: 1.07 – 1.59) for anxiety, 1.48 (95% CI: 1.17 – 1.88) for stress, 1.70 (95% CI: 1.32 – 2.18) for all three conditions, and 1.53 (95% CI: 1.25 – 1.87) for any two conditions.

Table 2. Adjusted odds ratios and their 95% Confidence intervals (CI) for mental health issues.

Variables	Depression	Anxiety	Stress	All three	Any two
Country (ref: Ghana)	AOR (CI)	AOR (CI)	AOR (CI)	AOR (CI)	AOR (CI)
Malawi	4.39 (3.28 – 5.89)	3.86 (2.81 – 5.29)	4.51 (3.33 – 6.11)	4.67 (3.41 – 6.41)	4.64 (3.46 – 6.23)
Mozambique	2.03 (1.55 – 2.67)	1.68 (1.27 – 2.22)	1.98 (1.46 – 2.69)	2.08 (1.51 – 2.85)	2.02 (1.54 – 2.66)
Nigeria	1.59 (1.30 – 1.95)	1.30 (1.07 – 1.59)	1.48 (1.17 – 1.88)	1.70 (1.32 – 2.18)	1.53 (1.25 – 1.87)

*All models were adjusted for gender, age group, marital status, educational level, ethnicity, religion, location of workplace, occupation, employment status, and having children (See Table 1 for the full variable list)

Discussion

The study highlights significant variations in mental health outcomes among the countries studied, with Malawi demonstrating the highest prevalence of mental health conditions. These differences align with existing research [29-31] which attribute disparities in mental health outcomes to factors such as socioeconomic conditions, access to healthcare, social support networks, and cultural attitudes toward mental health [32, 33]. The higher prevalence of mental health issues in Malawi may reflect economic hardship, food and job insecurity, and limited healthcare infrastructure[16, 17]. Additionally, cultural perceptions and the stigmas surrounding mental health may influence self-reported symptoms[20] further contributing to the disparities observed between Malawi and other countries, such as Ghana.

Analysis of mental health severity revealed high rates of moderate to severe conditions, especially for anxiety, with 27.7% of respondents experiencing ‘extremely severe’ anxiety similar to the findings from other studies[21, 22]. Although depression was common, it was generally less severe reflecting global trends[23] but also suggests that depressive symptoms are influenced by varying cultural and social factors across countries. The low rate of 'mild' depression may suggest underreporting of early symptoms or poor recognition[34], with individuals likely seeking treatment only when symptoms worsen[35]. In contrast, most (69.8%) of the respondents had stress levels within ‘normal’ limits stress levels suggesting that they are relatively more controllable[36, 37]. It is likely that, whereas anxiety is more closely associated with feelings of worry and fear, stress may be more dependent on particular situations like social and cultural expectations, economic conditions, and availability of healthcare services[38]. More so, stress may be influenced by coping mechanisms, social support, and workplace conditions, which can differ from one country to another[39]. The severity of mental health conditions was significantly higher in Nigeria, where 16.0% reported extreme anxiety. Although this contrasts with the findings of other studies [40, 41] which reported higher levels of extremely severe anxiety and is indicative of country-specific stressors such as economic and political instability, high levels of violence or conflict, or limited access to mental health services[17, 42] which may aggravate the mental health conditions.

Sociodemographic factors such as age, gender, and marital status also influenced mental health outcomes in line with existing literature[43, 44], with younger, unmarried females reporting higher prevalence due to life stressors and gendered expectations[45, 46]. In this study, students, 2807 (87.0%) and females, 1,850 (57.7%) were the largest subsets of the study population. Studies have shown that conditions such as depression, anxiety, and post-traumatic stress disorder are prevalent among African university students[47], with young women more likely to experience these issues[48, 49] due to additional risk factors like poverty, gender-based violence, and societal stigma associated with mental illness[50]. University-based studies in Kenya and South Africa reveal that gender-sensitive policies, mental health awareness, and campus support programs can reduce stigma and improve mental health outcomes for female students[51, 52].

Youth unemployment is a growing issue in many African countries, particularly impacting young adults' mental health[53]. Our findings also showed that respondents from urban areas were overrepresented among those reporting high mental health burdens, possibly due to urban stressors like overcrowding, housing instability, and limited access to healthcare indicating that mental health interventions should address these unique sociodemographic influences[54-56].

Our finding that Malawi has a higher prevalence of mental health disorders aligns with previous research [16, 17, 57, 58], which attributed this to factors such as poverty, food insecurity, limited healthcare infrastructure, and chronic stress. However, our study expands on this by providing a cross-national comparison and highlighting the unique challenges faced by each country. Residing in Malawi was associated with the highest risk for mental health disorders, with odds ratios ranging from 3.54 to 4.09, even after adjusting for sociodemographic variables. These results suggest that Malawi's mental health crisis is severe and ongoing, even after accounting for demographic factors. Mozambique showed a higher likelihood of depression and anxiety compared to Nigeria, though the impact was less severe than in Malawi. While mental health remains a concern in Mozambique, the lower odds suggest that the crisis may be less intense, possibly due to fewer or more mitigated socioeconomic stressors.

Moreover, comorbidity between depression, anxiety, and stress was common across the sample, with 51.3% of respondents experiencing two of these conditions, and over 25% affected by all three simultaneously. This comorbidity highlights the interconnectedness of these conditions [39], where one disorder can exacerbate the others. Mental health services, therefore, should adopt integrated treatment plans that effectively address multiple conditions simultaneously[59]. This may require collaboration among healthcare providers, including mental health professionals, primary care physicians, and social workers for more comprehensive care that tackles the various aspects of patients' mental health challenges[60]. The considerable overlap among mental health conditions indicates that recognizing and addressing issues at their initial stages could help avert the development of multiple co-occurring disorders.

Country-level analysis revealed distinct patterns in mental health outcomes, with Nigeria showing the highest prevalence of severe mental health disorders. These differences may also reflect varying economic or societal pressures in these countries, with Nigeria potentially experiencing more acute or chronic stressors that contribute to these mental health outcomes[61]. It is acknowledged that the larger sample size of Nigerian participants may contribute to the observed prominence of the country in the severe and extremely severe mental health categories. Conversely, Ghana's relatively favourable mental health outcomes, particularly in anxiety and stress, may reflect protective factors such as stronger social support[62] or the presence of effective coping mechanisms[63]. In addition, it has been noted that while Ghana struggles with under-resourced mental health services, recent policy improvements, such as the 2012 Mental Health Act and ongoing national mental health initiatives, have bolstered its infrastructure slightly[64]. Ghana's relative economic stability and stronger policy frameworks for mental health compared to Malawi may contribute to the differences observed in mental health statistics between the two countries.

Mozambique and Malawi showed elevated rates of two-condition prevalence, particularly in stress and anxiety, suggesting that while these countries face significant mental health challenges, the conditions may not frequently co-occur at extreme levels, as seen in Malawi. Malawi reported the highest rates of depression (72.3%), anxiety (81.4%), and elevated stress (50.6%), surpassing levels observed in other studies[23, 65]. In Mozambique, about 21.2% of adolescents experience psychosocial distress due to factors like bullying, violence, and hunger [66], highlighting the urgent need for robust mental health support in schools and communities

Additionally, cultural views on mental health, including stigma, may discourage individuals from seeking help early, resulting in delayed care until their conditions become severe[67]. Mozambique and Nigeria follow Malawi in the prevalence of two or all three conditions, though at lower rates. Mozambique's notable two-condition prevalence suggests factors that may limit the co-occurrence of all three disorders, while Nigeria's higher rates of individual conditions and slightly lower comorbidity imply that mental health disorders there may be more isolated rather than co-occurring.

The findings stress the need for targeted, context-specific mental health interventions in Sub-Saharan Africa. In Malawi, for example, where the mental health burden is severe, resources should prioritize accessible, community-based mental health programs that could be integrated into existing HIV/AIDS initiatives to leverage existing resources and address the co-occurrence of mental health issues and HIV/AIDS. In Nigeria, anti-stigma campaigns could be developed in collaboration with religious leaders and community organizations to reach a wider audience and address cultural beliefs about mental health and socioeconomic issues such as poverty and unemployment. Mozambique would benefit from strengthened mental health infrastructure and awareness programs, Ghana's comparatively positive mental health outcomes provide valuable insights that could inform strategies in higher-prevalence countries, particularly around community cohesion and resilience-building. More so, interventions should incorporate mental health literacy at an individual level, to support early detection of mental health issues.

Strengths and limitations of the study

This study's strengths include a large, diverse sample size and a cross-national focus on Sub-Saharan Africa (SSA). This provides valuable information on mental health across varied socio-economic and cultural backgrounds. The validated DASS-21 scale guaranteed reliable data, and the web-based survey enabled widespread reach. However, the cross-sectional design limits causal inference, and reliance on self-reported data may introduce respondent bias, particularly in populations with strong mental health stigma.

While the study's findings provide valuable insights into mental health disparities in SSA, the reliance on convenience sampling limits the generalizability of the results to the wider population. Future studies should consider employing probability sampling techniques to ensure greater representativeness. University students and staff tend to be younger and more educated than the general population, and this may influence their mental health experiences. Incorporating qualitative data from the survey (e.g., open-ended responses) in future studies could provide richer insights into the participants' experiences and perspectives on mental health.

Conclusions

The findings from this study suggest a strong link between a person's level of education and their risk for depression, anxiety, and stress. Those with higher levels of education may be better able to protect themselves from mental health challenges while individuals with lower levels of education may be more susceptible to mental health conditions. Furthermore, men are less likely than women to experience depression and stress. This may be because women face more pressure from social expectations and cultural norms. Though this study did not find a relationship between age and mental health, it is important to consider the limitations of the study population. Because many respondents reported experiencing multiple mental health issues at once, a more holistic treatment approach may be needed in this region. Overall, these findings highlight the critical mental health burden in SSA and underscore the need for increased access to mental health resources and targeted interventions in SSA such as community-based care in Malawi, stigma reduction interventions in Nigeria, infrastructure development in Mozambique, and resilience-building initiatives informed by Ghana’s successes.

Declarations

Funding: This research did not receive any funding.

Conflicts of interest/Competing interests: The authors have no financial disclosures to make and no conflict of interest.

Availability of data and material: The data that support the findings of this study are available on request from the corresponding author, ULO.

Authors' contributions: All authors made substantial contributions to the conception or design of the work; or the acquisition, analysis, or interpretation of data for the work.

Acknowledgment:

References

1. Ahmed YA, Anshur A, Abdishakur M, Sheik, Abdullahi A, Mohamed, et al. Prevalence of depression, anxiety, and stress among healthcare professionals in Mogadishu, Somalia. Somalia Int J Future Med Res. 2024;6(4):July-August. doi: 10.36948/ijfmr.2024.v06i04.24067.
2. Arias D, Saxena S, Verguet S. Quantifying the global burden of mental disorders and their economic value. EClinicalMedicine [Internet] 2022;54:101675. doi: https://doi.org/10.1016/j.
3. Jalali A, Ziapour A, Karimi Z, Rezaei M, Emami B, Kalhori RP, et al. Global prevalence of depression, anxiety, and stress in the elderly population: a systematic review and meta-analysis. BMC Geriatrics. 2024;24(1):809. doi: 10.1186/s12877-024-05311-8.
4. Jörns-Presentati A, Napp A-K, Dessauvagie AS, Stein DJ, Jonker D, Breet E, et al. The prevalence of mental health problems in sub-Saharan adolescents: A systematic review. PLOS ONE. 2021;16(5):e0251689. doi: 10.1371/journal.pone.0251689.
5. Kessler RC, Aguilar-Gaxiola S, Alonso J, Chatterji S, Lee S, Ormel J, et al. The global burden of mental disorders: An update from the WHO World Mental Health (WMH) Surveys. Epidemiologia e Psichiatria Sociale. 2009;18(1). doi: 10.1017/S1121189X00001421.
6. Liu Q, He H, Yang J, Feng X, Zhao F, Lyu J. Changes in the global burden of depression from 1990 to 2017: Findings from the Global Burden of Disease study. Journal of Psychiatric Research. 2020;126:134-40. doi: 10.1016/j.jpsychires.2019.08.002.
7. World Health Organisation. Depressive disorder (depression). 2023 [cited 2024 Nov 29]. Available from: https://www.who.int/news-room/fact-sheets/detail/depression.
8. Chaulagain A, Pacione L, Abdulmalik JO, Hughes P, Kopchak O, Chumak S, et al. WHO Mental Health Gap Action Programme Intervention Guide (mhGAP-IG): the first pre-service training study. International Journal of Mental Health Systems. 2020 Dec 29;14(1):47. doi: 10.1186/s13033-020-00379-2.
9. Faidas M, Stockton MA, Mphonda SM, Sansbury G, Hedrick H, Devadas J, et al. Stigma and discrimination faced by adolescents living with HIV and experiencing depression in Malawi. BMC Global and Public Health. 2024;2(1):39. doi: 10.1186/s44263-024-00072-3.
10. Choi M, Kirabo SJ, Lee EH, Lee YH. Differential trends and patterns of sociodemographic disparities in burden of mental disorders, substance use disorder and self-harm across age groups: Ecological study in 204 countries using the Global Burden of Disease Study 2024;10(3):e89.
11. Irfan M. Barriers to mental healthcare improvement in low and middle income countries Journal of Postgraduate Medical Institute. 2021;35(3):129-30.
12. Patel V. Mental health in low-and middle-income countries. British medical bulletin. 2007;81(1):81-96.
13. Meeks K, Peak AS, Dreihaus A. Depression, anxiety, and stress among students, faculty, and staff. Journal of American College Health. 2023;71(2):348-54. doi: 10.1080/07448481.2021.1891913.
14. Cortez CP. Prevalence and socio-demographic characteristics associated with depression, anxiety and stress among university students. Límite (Arica). 2024;19(16):1-14. doi: 10.4067/s0718-50652024000100216.
15. Pavllo M. The impact of sleep deprivation on symptoms of anxiety, depression, stress and on the quality of life in medical staff. European Psychiatry. 2024;67(S1):S772-S. doi: 10.1192/j.eurpsy.2024.1607.
16. Makombe T, Lewin PA, Fisher M. The Determinants of Food Insecurity in Rural Malawi: Implications for Agricultural Policy. 2010 July. Report No.
17. Zuberi A, Waqas A, Naveed S, Hossain MM, Rahman A, Saeed K, et al. Prevalence of Mental Disorders in the WHO Eastern Mediterranean Region: A Systematic Review and Meta-Analysis. 2021;12.
18. Hussain M, Rana DH, Ashraf R, Khurram S, Sindhu ZM. Exploring Cultural Factors in the Perception and Treatment of Depression: A Qualitative Investigation into the Influence of Cultural Beliefs and Values on the Experience Expression and Management of Depressive Symptoms. Journal of Population Therapeutics & Clinical Pharmacology. 2023:947-58. doi: 10.53555/jptcp.v30i19.3778.

19. Kadagidze L. Global Stigmas Surrounding Mental Health: Cultural and Ethnic Commonalities and Contributing Factors. Conference: International Texas Congress on Advanced Scientific Research and InnovationAt: Houston, Texas, USA. 2024.
20. Soares MR, Cameira MP, Abreu P, Pereira M. Cultural Diversity and Mental Health Care: A Case Study. European Psychiatry. 2024;67(S1):S522-S. doi: 10.1192/j.eurpsy.2024.1084.
21. Kithmini JPD, Kaarthiga S, Perera VBJ, Deshapriya HDS, Gamage CKW, Fernando NFJ. Depression, Anxiety, and Stress among Allied Health Sciences undergraduates: A Cross-Sectional Study at a Defence University in Sri Lanka. Journal of Multidisciplinary Studies. 2024;6(1).
22. Musfara DD, Widiyawati W, Fitrianur WL. Depression, Anxiety, Stress Correlated with Suicide Idea on Students of The Faculty of Health. Jurnal Ners dan Kebidanan (Journal of Ners and Midwifery). 2024;11(1):049-56. doi: 10.26699/jnk.v11i1.ART.p049-056.
23. Roy N, Paul KD, Tamanna SS, Paul AK, Almerab MM, Mamun MA. Prevalence and risk factors of depression, anxiety, and stress among the Bangladeshi construction workers: A cross-sectional study. PLOS ONE. 2024;19(8):e0307895. doi: 10.1371/journal.pone.0307895.
24. Covibond SH, Lovibond PF. Manual for the Depression Anxiety Stress Scales. 2 ed: Sydney, Psychology Foundation; 1995.
25. Oei TPS, Sawang S, Goh YW, Mukhtar F. Using the Depression Anxiety Stress Scale 21 (DASS-21) across cultures. International Journal of Psychology. 2013;48(6):1018-29. doi: 10.1080/00207594.2012.755535.
26. Osman A, Wong JL, Bagge CL, Freedenthal S, Gutierrez PM, Lozano G. The Depression Anxiety Stress Scales—21 (DASS-21): Further Examination of Dimensions, Scale Reliability, and Correlates. Journal of Clinical Psychology. 2012;68(12):1322-38. doi: DOI: 10.1002/jclp.21908.
27. Ng F, Trauer T, Dodd S, Callaly T, Campbell S, Berk M. The validity of the 21-item version of the Depression Anxiety Stress Scales as a routine clinical outcome measure. Acta Neuropsychiatrica. 2007;19(5):307-10. doi: DOI: 10.1111/j.1601-5215.2007.00217.x.
28. Lachenbruch PA, Lwanga SK, Lemeshow S. Sample Size Determination in Health Studies: A Practical Manual. Journal of the American Statistical Association. 1991;86(416):1149. doi: DOI: 10.2307/2290547.
29. Alshehri H, Alturkestani R, Hijan[...] B, Abdulghaffar HK. The prevalence of existential anxiety and its association with depression, general anxiety, and stress in Saudi university students. Middle East Current Psychiatry. 2024;31(1):47.
30. Habtu Y, Kumie A, Selamu M, Harada H, Girma E. Prevalence and determinants of occupational depression, anxiety, and stress among Ethiopian healthcare workers. Scientific Reports. 2024;14(1):21817. doi: 10.1038/s41598-024-72930-x.
31. Krishna Goud GS, P D. A Study on the prevalence of depression, anxiety, and stress in MBBS students. International Journal Of Scientific Research. 2024:25-6. doi: 10.36106/ijsr/5900947.
32. Kato JK. Cultural Attitudes towards Women in Technology in Uganda: Historical Factors and Contemporary Challenges. 2024.
33. Pandey DM. Access to the Healthcare in Remote Areas is Support for Poverty Reduction. International Journal of Social Science and Human Research. 2024;7(2). doi: 10.47191/ijsshr/v7-i02-65.
34. Sharma N, Chakrabarti S, Grover S. Gender differences in caregiving among family - caregivers of people with mental illnesses. World Journal of Psychiatry. 2016;6(1):7. doi: DOI: 10.5498/wjp.v6.i1.7.
35. Klineberg E, Biddle L, Donovan J, Gunnel D. Symptom recognition and help seeking for depression in young adults: a vignette study. Social psychiatry and psychiatric epidemiology 2011;46:495-505.
36. Rozhdestvenskiy VI, Titova VV, Gorkovaya IA, Ivanov DO, Aleksandrovich YS. Fundamental beliefs, as well as levels of depression, anxiety, and stress experienced by Russian students during the second wave of the COVID-19 pandemic. European Psychiatry. 2024;67(S1):S513-S. doi: 10.1192/j.eurpsy.2024.1066.
37. Yazid JB, Yusop YM. Association Between Socio-Demographic And Mental Health: A Study Of Depression, Anxiety, And Stress In Nursing Students. 2024.
38. Neyazi A, Mohammadi AQ, Razaqi N, Rahimi BA, Rahimi BA, Sifat S, et al. Health survey on anxiety, depression, and stress in Afghanistan: A large-scale cross-sectional survey study. 2024. doi: 10.21203/rs.3.rs-4149818/v1.

39. El-Ghitany EM, Ashour A, Elrewany E, Farghaly AG, Shata Z. Prevalence and predictors of depression, anxiety, and stress among refugees in Egypt. Journal of the Egyptian Public Health Association,. 2024;99(1):14. doi: 10.1186/s42506-024-00158-8.

40. Kumbet SJ, Oseni TIA, Mensah-Bonsu M, Damagum FM, Opare-Lokko EBA, Namisango E, et al. Common adolescent mental health disorders seen in Family Medicine Clinics in Ghana and Nigeria. . PLOS ONE. 2023;18(11):e0285911. doi: 10.1371/journal.pone.0285911.

41. Rubab T, Noureen S, Manzoor A. Evaluating the Societal Impact of Climate Change on Mental Health: Heat-Related Stress, Anxiety, and Depression in Vulnerable Populations. Contemporary Issues in Social Sciences and Management Practices. 2024;3(2):151-64. doi: 10.61503/cissmp.v3i2.169.

42. Agyepong I, Sewankambo N, Binagwaho A, Coll-Seck AM, Corrah T, Ezeh A, et al. The path to longer and healthier lives for all Africans by 2030: The Lancet Commission on the future of health in sub-Saharan Africa. The Lancet. 2017;390((10114)):2803–59. doi: https://doi.org/10.1016/S0140-6736(17)31509-X.

43. Ervin J, Milner A, Kavanagh A, King T. 1198 The gendered associations between poverty, marital status, and mental health in older Australians. . International Journal of Epidemiology. 2021;50(Supplement_1). doi: 10.1093/ije/dyab168.192.

44. Malek Mohammadi N, Rezaeisharif F, Bagheri N, Taheri Olyayie H, Sharifi M, Sharifi H. Prevalence of mobile phone addiction and poor mental health, and factors associated with mental health among medical students in Southeast Iran. BMC Psychiatry. 2024;24(1):552. doi: 10.1186/s12888-024-05985-9.

45. Bilodeau J, Beauregard N, Haines V, Quesnel-Vallée A. Work-family interface and mental health inequalities between women and men: A gendered exposure model across Canadian provinces. 2024. doi: https://doi.org/10.13140/RG.2.2.19570.93129.

46. Wang D, Yang M. Gendered mobility and activity pattern: implications for gendered mental health. Journal of Transport Geography. 2023;110:103639. doi: 10.1016/j.jtrangeo.2023.103639.

47. Julius B, Putteeraj M, Somanah J. A systematic review of university students' mental health in sub-Saharan Africa. Transformation in Higher Education. 2024;9. doi: 10.4102/the.v9i0.316.

48. Chigerwe M, Holm DE, Mostert E-M, May K, Boudreaux KA. Exploring issues surrounding mental health and wellbeing across two continents: A preliminary cross-sectional collaborative study between the University of California, Davis, and University of Pretoria. PLOS ONE. 2020;15(10):e0241302. doi: DOI: 10.1371/journal.pone.0241302.

49. Tsegay L, Tesfaye G, Ayano G. The Prevalence and Associated Factors of Suicidal Attempt Among Medical Students in Addis Ababa Ethiopia. Psychiatric Quarterly. 2021;92(1):193-205. doi: DOI: 10.1007/s11126-020-09790-x.

50. Muhia J, Nadine N. Youth Mentalhealth in the context of COVID-19 in the East and Southern Africa: A desk review. Harare Retrieved. 2021;11(15):2021.

51. Mthiyane N, Rapulana AM, Harling G, Copas A, Shahmanesh M. Effect of multi-level interventions on mental health outcomes among adolescents in sub-Saharan Africa: a systematic review. BMJ Open. 2023;13(10):e066586. doi: 10.1136/bmjopen-2022-066586.

52. Votruba N, Ziemann A, Grant J, Thornicroft G. A systematic review of frameworks for the interrelationships of mental health evidence and policy in low- and middle-income countries. Health Research Policy and Systems. 2018;16(1):85. doi: 10.1186/s12961-018-0357-2.

53. Olupona T, Jegede O, Keseke C, Akerele E. Mental Health Issues in Adolescents and Young Adult African Immigrants. Environment and Social Psychology. 2019;4(1). doi: 10.18063/esp.v4.i1.946.

54. Aldhahir A. Prevalence and sociodemographic factors of depression, anxiety and stress in Saudi Arabia: a survey of respiratory therapists. Frontiers in Psychiatry. 2024;15:1289456. doi: 10.3389/fpsyt.2024.1289456.

55. de Oliveira Viana MÁ, Porto É, dos Santos Dantas L, Soares Forte FD, D'Ávila Lins Bezerra Cavalcanti S, de Lima Targino Massoni AC. Depression, Anxiety, and Stress in Dental Students and Associations with Sociodemographic Variables and the Academic Environment. Trends in Psychology. 2024. doi: 10.1007/s43076-024-00359-2.

56. Silva GCBd, Barbosa GJM, Santiago MCdB, Santos ÍCd, Olinda RAd. Stress, Anxiety, and Depression in Dentistry Students: The Impact of the COVID-19 Pandemic and Sociodemographic

Factors. Pesquisa Brasileira em Odontopediatria e Clínica Integrada. 2024;24. doi: 10.1590/pboci.2024.097.
57. Hargraves JL, Cosenza C, Cleary PD. Measuring Access to Mental Health Services Among Primary Care Patients. Medical Care. 2024;62(8):559-6. doi: 10.1097/MLR.0000000000002029.
58. Hussain S, Pareek A, Singh M. Common Mental Disorders among individuals living in Poverty. 2024. doi: 10.25215/9173080780.08.
59. Felten CL, Smith KS, Aylesworth MB. An Integrated Approach to Address Perinatal Mental Health Within an Obstetrics Practice. Journal of Midwifery & Women's Health. 2024;69(5):778-83. doi: doi.org/10.1111/jmwh.13658.
60. Silvonen T. Service Providers' Perspectives of an Integrated Community Mental Health Service in the UK. Community Mental Health Journal. 2024. doi: 10.1007/s10597-024-01352-w.
61. Okirie AJ. Examining the effect of maintenance work on employees' physical and mental health, as well as their overall well-being, in industrial settings. Global Journal of Engineering and Technology Advances. 2024;19(3):131-42. doi: 10.30574/gjeta.2024.19.3.0108.
62. Agbozo F, Amobaa ND, Runge-Ranzinger S, Grys P. 1072 Mental health difficulties among adolescents in Ghana, coping mechanisms and the support systems: A mixed-methods study. . International Journal of Epidemiology, . 2021;50(Supplement 1). doi: https://doi.org/10.1093/ije/dyab168.012.
63. Schweitzer R, Melville F, Steel Z, Lacherez P. Trauma, Post-Migration Living Difficulties, and Social Support as Predictors of Psychological Adjustment in Resettled Sudanese Refugees. Australian & New Zealand Journal of Psychiatry. 2006;40(2):179-87. doi: 10.1080/j.1440-1614.2006.01766.x.
64. Awortwe V, Daivadanam M, Adjorlolo S, Olsson EM, Coumoundouros C, Woodford J. Prevalence and social determinants of anxiety and depression among adults in Ghana: a systematic review and meta-analysis protocol. BMJ Open. 2024;14(4):e081927. doi: 10.1136/ bmjopen-2023-081927.
65. Samsoor AA, Stanikzai MH. Depression, Anxiety and Stress Symptoms among Afghan Healthcare Workers: A Cross-Sectional Analytical Study. Indian Journal of Occupational and Environmental Medicine. 2024;28(2):154-8. doi: 10.4103/ijoem.ijoem_34_24.
66. Amu H, Seidu A-A, Agbemavi W, Afriyie BO, Ahinkorah BO, Ameyaw EK, et al. Psychosocial distress among in-school adolescents in Mozambique: a cross-sectional study using the Global School-Based Health Survey data. Child and Adolescent Psychiatry and Mental Health. 2020;14(1):38. doi: 10.1186/s13034-020-00344-4.
67. Jeffrey J, Puentespina S, Decatoria J, Alave A. Pandemic Emotional Impact, Resilience, Stigma, Help-Seeking, And Mental Health Condition Of College Students: A Path Analysis * Pandemic Emotional Impact, Resilience, Stigma, Help-Seeking, And Mental Health Condition Of College Students:. 2023.

Figure Legends

Figure 1: Flowchart of Respondent Selection Process in Study Analysis

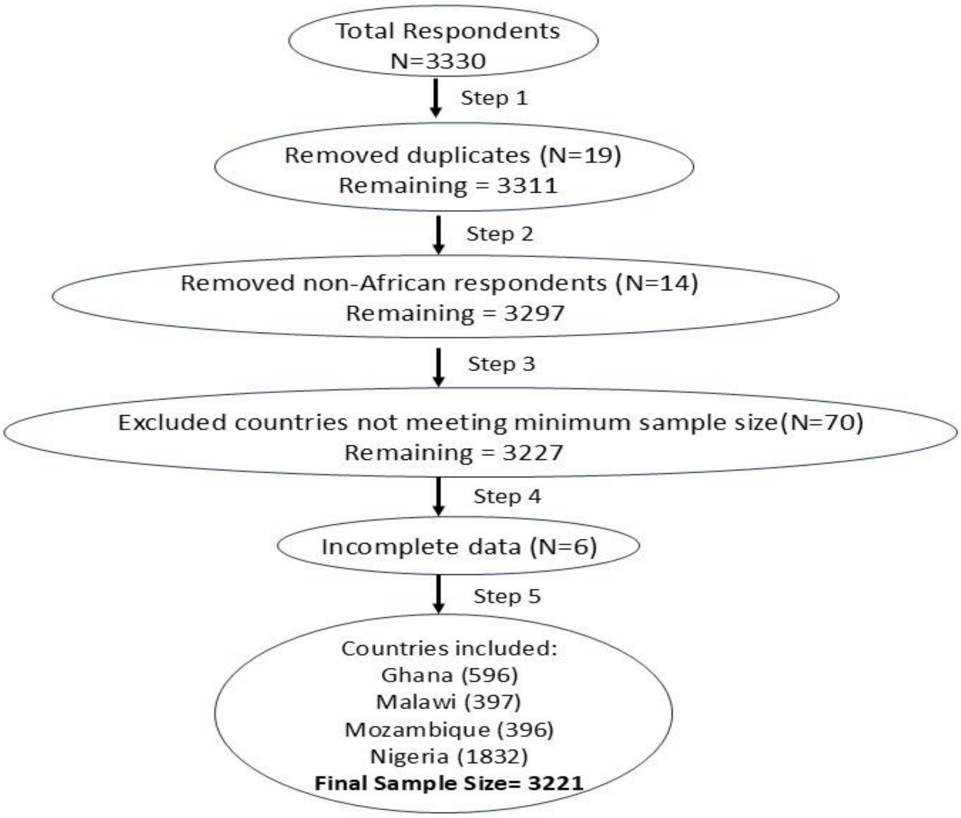

Figure 2: (Top) Overall and (Bottom) country-specific severity distribution of depression, anxiety, and stress among staff and students of African universities.

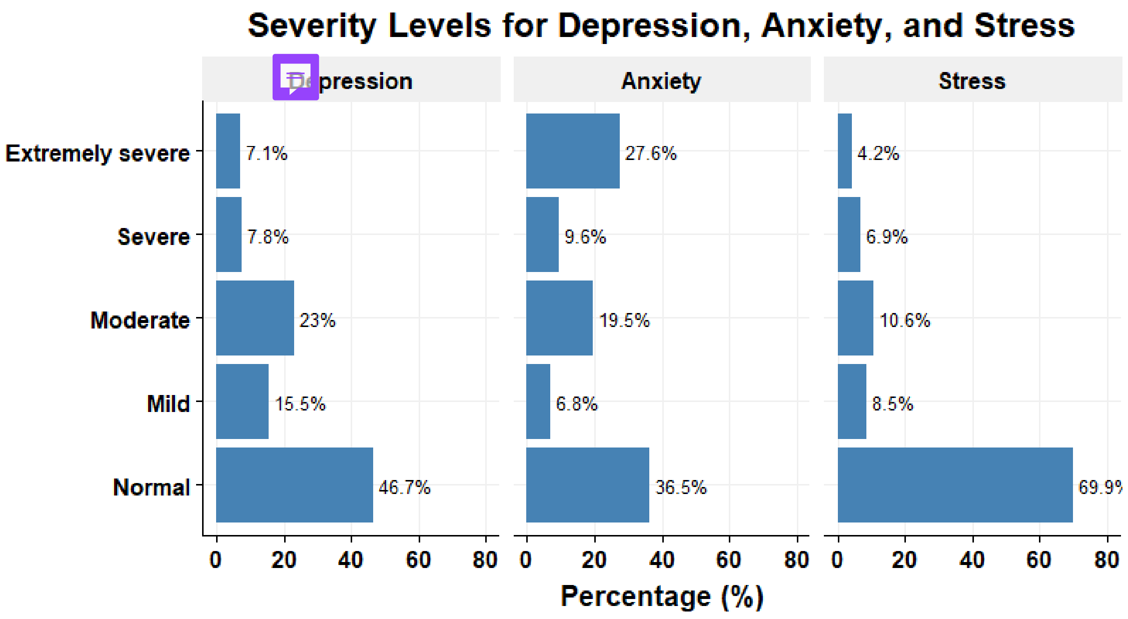

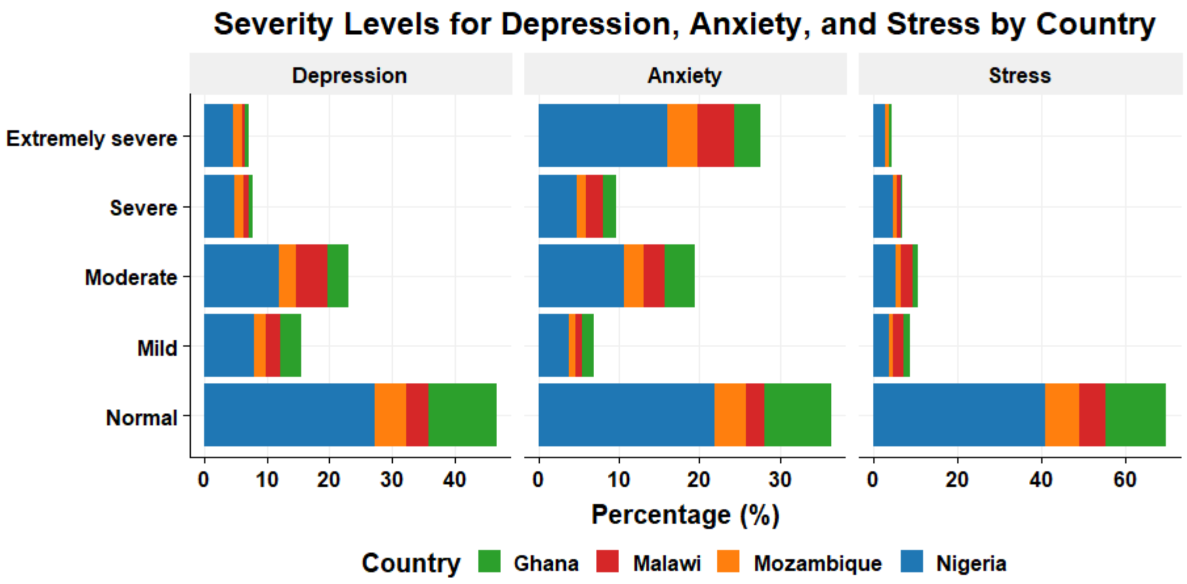

Figure 3. Country-level risk associations with mental health conditions—specifically, depression, anxiety, stress, and combinations of these conditions—with Ghana as the reference category. The box size is proportional to the OR.

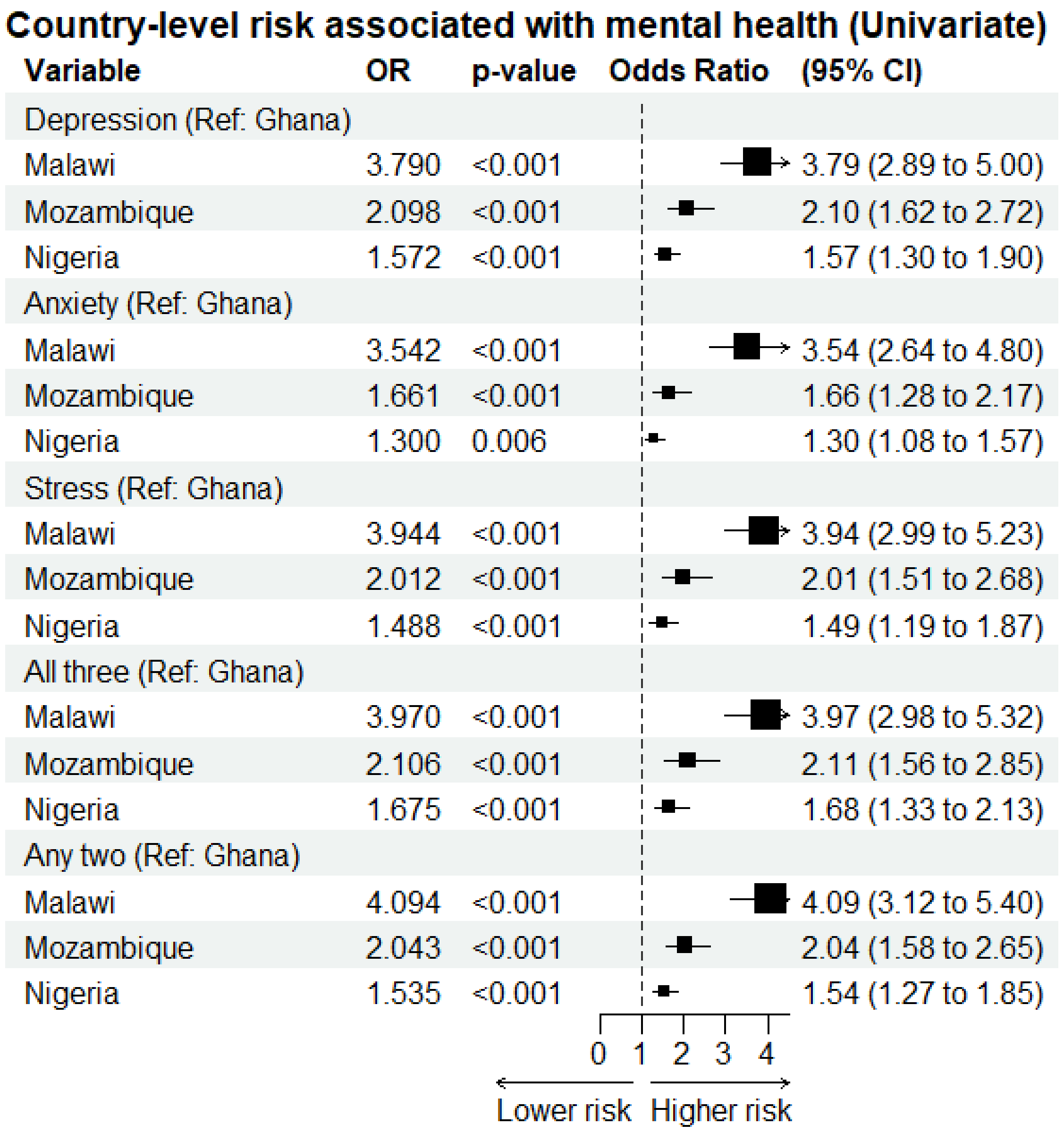

Supplementary Files

Supplementary File 1:

Supplementary File 2:

Click here to access/download
Supporting Information - Compressed/ZIP File Archive
Supplemenatry Files Cross National Variation on MH.zip

PONE-D-24-56128
Cross-National Variations in Mental Health: A Cross-sectional Study on Depression, Anxiety, and Stress in sub-Saharan Africans
Dr Uchechukwu L. Osuagwu

Dear Dr. Osuagwu,

We've checked your submission and before we can proceed, we need you to address the following issues:

1. Please complete all items on the Human Participants Research Checklist that are relevant for your submission, by following this link: http://journals.plos.org/plosone/s/file?id=dc11/PLOSOne_Human_Subjects_Research_Checklist.docx (Contact us at plosone@plos.org if you cannot access the document.)

Response: Done

There may be overlap between the checklist items and other queries listed below; please address any duplicated queries both in your response email and on the checklist itself. Upload the completed Human Participants Research Checklist as file type "Other" when you re-submit your manuscript. This document is for internal journal use only and will not be published if your article is accepted. The requested information will help us to assess whether your submission complies with PLOS ONE's policies and adheres to applicable reporting standards. Note that your manuscript may be rejected if you provide incomplete or inadequate responses to the checklist questions and that changing the
'Section/Category' of your article does not affect this requirement.

2. Please provide additional details regarding participant consent. In the ethics statement in the Methods and online submission information, please ensure that you have specified what type you obtained (for instance, written or verbal, and if verbal, how it was documented and witnessed). If your study included minors, state whether you obtained consent from parents or guardians. If the need for consent was waived by the ethics committee, please include this information.

Response: Done. Line 241-245

Once you have amended this/these statement(s) in the Methods section of the manuscript, please add the same text to the "Ethics Statement" field of the submission form (via "Edit Submission").

Response: Done

For additional information about PLOS ONE ethical requirements for human subjects research, please refer to http://journals.plos.org/plosone/s/submission-guidelines#loc-human-subjects-research.

We've returned your manuscript to your account. Please resolve these issues and resubmit your manuscript within 21 days. If you need more time, please email the journal office at plosone@plos.org. We are happy to grant extensions of up to one month past this due date. If we do not hear from you within 21 days, we will withdraw your manuscript.

Please log on to PLOS Editorial Manager at https://www.editorialmanager.com/pone/ to access your manuscript. You will find your manuscript in the 'Submissions Sent Back to Author' link under the New Submissions menu. Be sure to remove your previous manuscript file if you are uploading a new file in response to these requests. After you've made the changes requested above, please be sure to

view and approve the revised PDF after rebuilding the PDF to complete the resubmission process.

We are requesting these changes to comply with the PLOS ONE submission guidelines (https://journals.plos.org/plosone/s/submission-guidelines). Please note that we won't send your manuscript for review until you have resolved the above requests.

Thank you for submitting your work to PLOS ONE and supporting our mission of Open Science.

Kind regards,

Baltimore Tabios Bejosano
PLOS ONE
