# Supplementary material for: Cross-National variations in mental health: A cross-sectional study on depression, anxiety, and stress among university staff and students in Sub-Saharan African
Source: PLoS One. 2025 Jun 3;20(6):e0322163. doi: 10.1371/journal.pone.0322163 (PMC12133007; doi:10.1371/journal.pone.0322163)
Supplement: Attachment — Submitted filename: Response to comments.docx [file pone.0322163.s002.docx]

PONE-D-24-56128
Cross-National Variations in Mental Health: A Cross-sectional Study on Depression, Anxiety, and Stress in sub-Saharan Africans
Dr Uchechukwu L. Osuagwu

Dear Dr. Osuagwu,

We've checked your submission and before we can proceed, we need you to address the following issues:

1. Please complete all items on the Human Participants Research Checklist that are relevant for your submission, by following this link: [http://journals.plos.org/plosone/s/file?id=dc11/PLOSOne_Human_Subjects_Research_Checklist.docx](https://url.au.m.mimecastprotect.com/s/RH62C1WLKMHM8pWMAu1uvIV5pbB?domain=journals.plos.org) (Contact us at plosone@plos.org if you cannot access the document.)

Response: Done

There may be overlap between the checklist items and other queries listed below; please address any duplicated queries both in your response email and on the checklist itself. Upload the completed Human Participants Research Checklist as file type “Other” when you re-submit your manuscript. This document is for internal journal use only and will not be published if your article is accepted. The requested information will help us to assess whether your submission complies with PLOS ONE’s policies and adheres to applicable reporting standards. Note that your manuscript may be rejected if you provide incomplete or inadequate responses to the checklist questions and that changing the
‘Section/Category’ of your article does not affect this requirement.

2. Please provide additional details regarding participant consent. In the ethics statement in the Methods and online submission information, please ensure that you have specified what type you obtained (for instance, written or verbal, and if verbal, how it was documented and witnessed). If your study included minors, state whether you obtained consent from parents or guardians. If the need for consent was waived by the ethics committee, please include this information.

Response: Done. Line 241-245

Once you have amended this/these statement(s) in the Methods section of the manuscript, please add the same text to the “Ethics Statement” field of the submission form (via “Edit Submission”).

Response: Done

For additional information about PLOS ONE ethical requirements for human subjects research, please refer to [http://journals.plos.org/plosone/s/submission-guidelines#loc-human-subjects-research](https://url.au.m.mimecastprotect.com/s/KlenC2xMLXfp7kyp2tXC2I5Q19I?domain=journals.plos.org).

We've returned your manuscript to your account. Please resolve these issues and resubmit your manuscript within 21 days. If you need more time, please email the journal office at plosone@plos.org. We are happy to grant extensions of up to one month past this due date. If we do not hear from you within 21 days, we will withdraw your manuscript.

Please log on to PLOS Editorial Manager at [https://www.editorialmanager.com/pone/](https://url.au.m.mimecastprotect.com/s/wHheC3QNM3TpZmNpgtEFLIQQjko?domain=editorialmanager.com) to access your manuscript. You will find your manuscript in the 'Submissions Sent Back to Author' link under the New Submissions menu. Be sure to remove your previous manuscript file if you are uploading a new file in response to these requests. After you've made the changes requested above, please be sure to view and approve the revised PDF after rebuilding the PDF to complete the resubmission process.

We are requesting these changes to comply with the PLOS ONE submission guidelines ([https://journals.plos.org/plosone/s/submission-guidelines](https://url.au.m.mimecastprotect.com/s/0WsLC4QON2TBrJkB2i3H5I4duNp?domain=journals.plos.org)). Please note that we won't send your manuscript for review until you have resolved the above requests.

Thank you for submitting your work to PLOS ONE and supporting our mission of Open Science.


Kind regards,

Baltimore Tabios Bejosano
PLOS ONE
