# Supplementary material for: Cross-National variations in mental health: A cross-sectional study on depression, anxiety, and stress among university staff and students in Sub-Saharan African
Source: PLoS One. 2025 Jun 3;20(6):e0322163. doi: 10.1371/journal.pone.0322163 (PMC12133007; doi:10.1371/journal.pone.0322163)
Supplement: Attachment — Submitted filename: Response to Reviewers comments.docx [file pone.0322163.s003.docx]

**Responses to Reviewers’ Comments**

Dear Editor and Reviewers,

Thank you for the time and effort invested in reviewing our manuscript. We appreciate your insightful comments and constructive suggestions, which have helped us improve the quality and clarity of our work. Below, we provide detailed responses to each of the reviewers’ comments. Changes made in the manuscript in response to these comments are highlighted for clarity.

We hope that the revised version of our manuscript meets your expectations and addresses all the concerns raised.

| **S/N** | **Line Number** | **Academic Editor’s Comment** | **Response to Reviewer’s Comment** |
| --- | --- | --- | --- |
| **Academic Editors comments** | | | |
|  | Line 92 – 93 | This was not part of your results. How did the authors come to this conclusion? | We have revised the sentence to read: ‘The findings emphasize the importance of considering contextual factors, such as education levels, gender, and country of origin, in understanding mental health risks’. |
|  | Line 134 | Methods:– “across several SSA countries”. Comment – if only 4 countries were used, why not indicate “across 4 SSA counties”? Using SSA may be misleading since there is specific number of countries that have participated. | The study initially involved participants from several SSA countries. However, for the analysis, only data from four countries met the minimum sample size requirement. However, we have revised this section to four SSA countries |
|  |  | Please include “subsection settings” and outline key socioeconomic characteristics of the countries. | A subsection ‘settings’ has been added on the sociodemographic of the countries. |
|  | Lines 182 – 183 versus  Lines 186 – 188. | Inclusion and exclusion criteria – this section is confusing. Please review The two statements were contradictory. | The statement has been revised to read “Survey responses from individuals who were not originally from SSA but were currently studying or working in the region were excluded from the analysis. Additionally, responses originating from identical Internet Protocol addresses and exhibiting similar demographic characteristics, such as age and gender, were considered duplicate entries. In such cases, only the most complete entry was retained for analysis. Participants who provided informed consent were included.” |
|  |  | Results: Please review Plosone guidelines about figures, captions and placement. All figures captions should be labelled in the text where they were firstly mentioned. Submission Guidelines \| PLOS ONE | The caption of the figures has been labelled in the text where they were first mentioned. |
|  | Line 471 | Acknowledgement:– Acknowledgement statement is missing | A statement has been included in the acknowledgment section “The authors would like to express their heartfelt gratitude to all respondents for their valuable time and participation, which contributed significantly to the success of this study.” |
|  |  |  |  |
|  | **Line Number** | **Reviewer 1 Comments** | **Response to Reviewer’s Comment** |
|  |  | The title should be made to reflect the subpopulation- staff and students, not looking like a community survey. | The title has been revised to read “Cross-National Variations in Mental Health: A Cross-sectional Study on Depression, Anxiety, and Stress among University staff and students in sub-Saharan African” |
|  |  | The introduction did highlight much on literatures that focused on higher institution population as depicted in their objectives. There is no adequate rationale for this study. Authors should take note. | The introduction has been revised to focus on higher institution population with supporting literature. |
|  |  | The study design was merged with study procedure. | The study design and procedure have been separated |
|  |  |  |  |
|  | **Line Number** | **Reviewer 2 Comments** | **Response to Reviewer’s Comment** |
|  | Line 122: | Introduction: The introduction section is thorough, the authors demonstrating a good understanding of the global and local burden of common mental health conditions. However, I have made the following observations: - The statement, “The current body of literature frequently neglects the role of cross-cultural differences among countries” should be supported by a reference. | References have been provided. |
|  | Line 142  Line 166 | Methods: -The authors noted that the questionnaire was translated and administered in Portuguese and English languages. The authors should have also translated the questionnaire to French, as one of the most common official languages in African countries. Perhaps this could have mitigated the non-response or incomplete responses from some country participants. The authors noted that the DAS-21 was previously validated and pre-tested in their study. It will be helpful to provide the Cronbach’s alpha estimates. | About the translation (Line 142), there were no translations to French language because of cost constraints and lack of representation in the group.  Line166: A reliability of the survey tools was confirmed with sufficient Cronbach’s alpha coefficients for the DASS-21, used to measure depression, anxiety, and stress. “The DASS-21 scale showed a reliability coefficient of 0.942, with a total of 21 items. The subscales for depression, anxiety, and stress had reliability coefficients of 0.806, 0.873, and 0.871, respectively, with each subscale containing 7 items.” |
|  |  | Results: - Figure 2: The caption should be below the figure. The authors should correct this. | The figure 2 has the caption below. |
|  |  | Table 1: The category “Islamic” under religion should be “Islam” | “Islamic” has been revised to “Islam”. |
|  | Line 257 | In the statement, “Additionally, 83.7% (n=2639) of respondents indicated that they did not have children”, the proportion should be 81.9% (2639/3221) | Thank you for the comment; however, the proportion we provided is the correct one since we had 3152 responses for this statement as shown in the table instead of 3221. |
|  | Line 263 | Prevalence and distribution of severity of mental health conditions: I suggest the authors present the prevalent estimates of the mental health conditions (depression, anxiety and stress) and their corresponding Confidence Intervals, by country, in a table. | A table has been inserted for the prevalences and confidence intervals, and the sections shortened to minimise repetition. |
|  | Line 290 | The statement, “For depression and anxiety, a higher percentage of Nigerians reported severe (4.9% and 4.7%) and extremely severe (4.6% and 16.0%) depression, while Ghanaians reported notably lower proportions, particularly in the severe anxiety category (1.6%)” should be corrected by replacing “depression” with “conditions”. | The “depression” has been replaced with “conditions”. |
|  | Line 286 | “classified as ‘normal’ (N = 2254, 69.9%)” the letter N should be lower case (n) for consistency. The authors should correct this. | Thank you, “N” has been replaced with “n” |
|  | Line 308 | For clarity and consistency, I suggest the authors present the Odds Ratios (ORs) with the 95% Confidence Intervals (CIs) together, e.g., 4.09 (95% CI: 3.12 to 5.40) in Malawi, 2.04 (95% CI: 1.58 to 2.65) in Mozambique, and 1.54 (95% CI: 1.27 to 1.85) in Nigeria. | Thank you, the statement has been revised to ensure consistency. “In comparison, those in Mozambique had odds ratios (OR) of 2.10 (95% CI: 1.62 to 2.72) and Nigeria, 1.57 (95% CI: 1.30 to 1.90).” |
|  | Line 430 | Discussion -: There should be a full stop after the sentence, “Mozambique would benefit from strengthened mental health infrastructure and awareness programs”. | A full stop has been inserted. |
|  | Line 450 | Conclusions. “The findings from this study suggest a strong link between a person's level of education and their risk for depression, anxiety, and stress”. These conclusions are not supported by the presented results. The authors should review this. | The conclusion has been revised to read “This cross-sectional study highlights significant cross-national variations in the prevalence of depression, anxiety, and stress among university staff and students in sub-Saharan Africa. The findings emphasize the importance of considering contextual factors, such as education levels, gender, and country of origin, in understanding mental health risks. Many respondents reported experiencing multiple mental health issues simultaneously, suggesting the need for a more holistic treatment approach in the region. Overall, these findings underscore the critical mental health burden in sub-Saharan Africa and the need for increased access to mental health resources and targeted interventions, such as community-based care in Malawi, stigma reduction initiatives in Nigeria, infrastructure development in Mozambique, and resilience-building strategies informed by Ghana’s successes.” |
|  |  |  |  |
|  | **Line Number** | **Reviewers’ Comments on the PDF Document attached** | **Response to Reviewer’s Comments** |
| 1 | Line 2-3 | The title is generalized, while the work focuses on mental health outcomes in a sub-population (Higher institutions and staff). Please, re-write the title to reflect the sub-population studied | The title has been rewritten to reflect the sub-population studied. It now reads, ‘**Cross-National Variations in Mental Health: A Cross-sectional Study on Depression, Anxiety, and Stress among University staff and students in sub-Saharan African** |
| 2 | Lines 92-93 | This was not part of your results. How did the authors come about this conclusion? | This has been expunged and the conclusion rewritten to reflect only the findings of the study. The conclusion now reads, ‘This study found significant differences in mental health outcomes between participants from Ghana, Malawi, Mozambique, and Nigeria. Malawi had the greatest risk for depression, anxiety, stress, and combinations of those conditions while Ghana reported the lowest risks. The findings emphasize the importance of considering contextual factors, such as education levels, gender, and country of origin, in understanding mental health risks. Overall, these findings underscore the critical mental health burden in sub-Saharan Africa and the need for increased access to mental health resources and targeted interventions.’ |
|  | Lines 100-107  Lines 112-115;  Lines 120-122;  Line 125  Lines 126-127;  Lines 127-129 | Line 100-107: This gave a global burden of those mental health outcomes you studied. Now what of the localized burden in the university sub-population across SSA? Of which your methodology and results focused on. Line 112-115: This is the only statement talking about what the authors aimed to study, in line with their methodology. So please re-write the Introduction to centre majorly on this theme. Line 120-122. These two phrases in this sentence contradict each other or are simply saying the same thing. Therefore, the authors should find a rationale for this study. Line 125: This study results did not fill this gap. Rephrase the entire sentence and even the preceding sentence. Or delete them entirely. Line 126-127: Yes, that is what the study did, across different higher institutions in those countries studied. Line 127-129: Not at all. The result did show this aim. Authors should rephrase the aim or objectives to align with the results. Is like working backward, from results to intro. | The entire introduction has been re-written to address all the reviews made. Please find below the revised Introduction.  **Introduction**  Mental health disorders, including depression, anxiety and stress, are a growing public health concern, with significant global prevalence [1, 2] which varies across populations [3, 4]. For example, the global incidence of depression increased from 172 million cases in 1990 to 258 million in 2017, marking a 49.86% rise [5, 6]. According to the World Health Organization[7], approximately 280 million people worldwide suffer from depression, a condition that disproportionately affects women and is often linked to economic burdens, stigma, discrimination, and social exclusion[8, 9][.](https://www.zotero.org/google-docs/?broken=vxFUpv) Mental health disorders affect individuals across all ages, genders, and socioeconomic backgrounds, exerting a widespread impact across diverse geographic and demographic groups[10]. In sub-Saharan African (SSA) countries, the burden of depression, stress, and anxiety in the university sub-population has been shown to be high (Adeleke et al., 2005; Farrau et al.,2024; Malebana et al., 2019) highlighting the need of addressing this group to establish solutions for their wellbeing.  Students and staff in universities within under-resourced educational systems in low- and middle-income countries are especially susceptible to mental health challenges, as they contend with distinctive stressors such as heavy academic workloads and intense pressure to perform[13-15]. Researching these groups offers valuable insights into their mental health challenges, enabling targeted interventions to improve health, academic performance, and workplace productivity, with benefits extending to society at large.  However, the current body of literature (Sala-Wright et al, 2024; Adebayo et al, 2024) frequently neglects the role of cross-cultural differences among the university sub-population across in SSA countries, leading to a significant gap in understanding. By examining the influence of country of origin on the prevalence and severity of mental health conditions such as depression, anxiety, and stress among University staff and students across SSA countries, this study aims to bridge the existing knowledge gap. Specifically, the objectives of this study include determining the prevalence of depression, anxiety, and stress among University staff and students across different sub-Saharan African countries, identifying significant cross-national variations and assessing the role of demographic variables in influencing mental health outcomes across these SSA countries. |
|  | Line 136-138 | Delete. Move it to study procedure section if you wish. | Moved to study procedure section. |
|  | Line 140 | Delete. | Deleted |
|  | Line 141-143 | Move this to study procedure | Moved to study procedure |
|  | Line 146-153 | Move this to study procedure section, please. | Moved to study procedure |
|  | Line 161-163 | Delete this from this section. | Deleted from the Questionnaire section and taken to the Study Procedure section. |
|  | Line 178-179 | You may state the changes or revision | ?? |
|  | Line 200-214 | Summarize the outcome variables, Predictor variables and Covariates move them under Data analysis. | Outcome variables, Predictor variables and Covariates have been summarized and moved under Data analysis. The summary reads,  The study utilized the DASS-21 survey to measure three outcome variables—depression, anxiety, and stress—which were analyzed as categorical variables based on classification thresholds. Mental health conditions were further dichotomized into binary categories: 0–9 (“No depression”) and 10+ (“Major depression”) for depression; 0–7 (“No anxiety”) and 8+ (“Anxiety”) for anxiety, and 0–14 (“No stress”) and 15+ (“Stress”) for stress. Predictor variables included country of origin, focusing on Ghana, Nigeria, Malawi, and Mozambique due to sufficient sample sizes. Covariates encompassed sociodemographic factors such as age, gender, marital status, education level, faculty category (student, academic staff, non-academic staff), religious affiliation, level of study, rurality, and country of residence. |
|  | Line 204 | The authors did not categorize this based on severity but on Present/absent. | This has been revised to read “based on whether present or absent” |
|  | Line 221 | OR calculation is a bivariate and not a univariate analysis | Replaced with bivariate |
|  | Line 225-228 | Delete not necessary | Deleted: ‘This approach accounted for the influence of these factors on the relationship between country of origin and mental health outcomes. Adjusted odds ratios (AORs) and their 95% confidence intervals (CIs) were derived from the adjusted multivariable logistic regression model to quantify the relationships. Interaction terms were not included in the model due to the complexity of the data’ |
|  | Line 233-234 | Delete | Deleted: "Do you consent to voluntarily participate in this survey?" Consent was implied when participants selected the "Yes" option. |
|  | Line 245-246 | Delete | Deleted: In addition, the university granted a waiver for obtaining assent due to the minimal risk associated with the study design. |
|  | Line 261 | Make the row for total bold | Row for Total made bold |
|  | Line 264 | State the table or Fig of these prevalences | ??? |
|  | Line 267 | Remove these % in the CI | % in the CI removed |
|  | Line 288 | This should be Fig III.  Not bottom and top labeling. Please also label your figures in the table and figure section for clarity. | ??? |
|  | Line 297 | Replace with Bivariate | Replaced with Bivariate |
|  | Line 298 | Figure 3 should be Fig IV | ??? |
|  | Line 309-311 | Delete, since this not a Discussion section. | Deleted: Malawi consistently showed the highest odds of mental health conditions compared to Ghana, with ORs ranging from 3.54 to 4.09 for different mental health outcomes. |
|  | Line 312 | Please, what exactly the statistical analysis that was carried out?  You mean- Multivariate logistic analysis? | ??? |
|  | Line 314 | The result write-up is just a photocopy of the table, which is not ideal. Authors should summarize the result text in not more than 5-6lines | Table 2 write-up summarized: It now reads “Table 2 presents the adjusted odds ratios (AORs) for mental health issues across the countries studied after adjusting for socio-demographic variables (See Table S1 for the full variable list).  Malawi and Nigeria exhibited the highest and lowest risks respectively of mental health issues compared to Ghana. Specifically, individuals in Malawi were 4.39 times more likely to be depressed, 3.86 (95% CI: 2.81 – 5.29) more likely to experience anxiety, and 4.51 times more likely to experience stress. They were also 4.67 times more likely to experience all three conditions simultaneously and 4.64 times more likely to face any two conditions. |
|  | Line 319 | Delete, this is not Discussion | Deleted : ‘compared to respondents from Ghana’ |
|  | Line 331-333 | Delete | Deleted:  ^*^All models were adjusted for gender, age group, marital status, educational level, ethnicity, religion, location of workplace, occupation, employment status, and having children (See Table 1 for the full variable list)’ |
|  | Line 336 | Correct this: among the countries | Corrected: The line now reads “ among the higher institutions in the countries studied |
|  | Line 364-365 | The authors used their frequency tally result to compare with bivariate analytical results of previous literatures. | New literature added to enable comparism:  Madhuri NK, Devulapally Y, Priyanka S, Shirisha J. Assessment of mental health status in male and female medical students of 1st-year MBBS. National Journal of Physiology, Pharmacy and Pharmacology. 2023;13(6):1158-60. doi: 10.5455/njppp.2023.13.11514202204112022  Gomes JC, Ponnamperuma L, Hettiarachchi A. A Comparison of Mental Well-being among Female Undergraduates in Sri Lanka based on Relationship Status. SLIIT Journal of Humanities and Sciences. 2023 Jun 30;4(1). |
|  | Line 372-373 | There is link this statement about unemployment and the rest of the statements in this paragraph.?? | The statement has been expunged. |
|  | Line 373-374 | Please, where is this result in your study? | The result has been removed as it is not part of the study. |
|  | Line 378-379 | This is not in your result | Statement has been expunged to keep discussion within the limits of the findings of the study. |
|  | Line 380-382 | This is not in your result, as well.  Authors should discuss their findings in comparison with others. | Statement has been expunged to keep discussion within the limits of the findings of the study. |
|  | Line 380-382 | Repetition of what was earlier stated in line 377. Delete | Deleted. |
|  | Line 426-427 | This is never part of your findings. | Deleted |
|  | Line 449-451 | The results of this study never revealed so. | Deleted. |
|  | Line 453-456 | Delete. This is not a conclusion idea. | Deleted |
|  | Line 460-461 | Delete this because your work did not produce those deficits and strengths in the respective countries. | Deleted  The entire Conclusion has been rewritten to reflect the findings of the study |
